# Identification of Arbuscular Mycorrhizal Fungal Isolates using MiSeq Sequencing

**DOI:** 10.1264/jsme2.ME25040

**Published:** 2025-11-14

**Authors:** Ryo Ohtomo

**Affiliations:** 1 Institute for Agro-Environmental Sciences, NARO, 3–1–3 Kannondai, Tsukuba, Ibaraki, 305–8604 Japan

**Keywords:** arbuscular mycorrhizal fungi, culture collection, ribosomal RNA region, high-throughput sequence

## Abstract

The standardization of strain identification methods is essential for effectively managing the genetic resources of arbuscular mycorrhizal (AM) fungi. Due to their highly polymorphic rRNA sequence and multinucleate nature, conventional strain identification using a single sequence of the rRNA region is often inadequate for these fungi. Therefore, the present study exami­ned the use of genetic diversity information obtained through high-throughput sequencing to improve the strain identification of AM fungal isolates. Five previously reported primer pairs were used to amplify a portion of the rRNA region from DNA extracted from AM fungal spores, which was then sequenced using the Illumina MiSeq platform. The majority of amplicon sequence variants (ASVs) matched the same strain as the source isolate. A cluster anal­ysis indicated that strains of the same species generally grouped together, demonstrating the method’s effectiveness for species-level identification. Furthermore, a phylogenetic anal­ysis revealed some strain-specific ASVs that may be valuable for differentiating between different strains within the same species. Based on these results, it is feasible to develop a reliable identification protocol for AM fungal isolates using MiSeq sequencing.

Arbuscular mycorrhiza (AM) is the most ubiquitous plant-microbe interaction in the terrestrial ecosystem. More than 80% of land plants form symbiotic relationships with AM fungi (Smith and Smith, 2011), and this symbiosis has wide-ranging functions. It enhances the uptake of soil nutrients and water by host plants, particularly phosphate (Ezawa and Saito, 2018; Smith *et al.*, 2004; Karandashov and Bucher, 2005) and nitrogen (Tanaka and Yano, 2005; Leigh *et al.*, 2009; Wang *et al.*, 2020). Symbiosis has also been shown to alleviate environmental stresses, such as drought (Khalvati *et al.*, 2005; Begum *et al.*, 2019) and heavy metal pollution of the soil (Yao *et al.*, 2014; Chen *et al.*, 2018), and mitigate damage caused by pests and insects (Akhtar and Siddiqui, 2008; Jung *et al.*, 2012). Furthermore, symbiosis plays important roles in environmental sustainability, such as stabilizing soil aggregates (Nichols, 2008) and reducing soil nutrient loss (Cavagnaro *et al.*, 2015). Fungi connect different plant individuals to share nutrients and information among their common hosts (van der Heijden and Horton, 2009; Johnson and Gilbert, 2015; Sharifi and Ryu, 2021). This symbiotic system has recently been considered important from the viewpoint of the mitigation of global warming by enhancing carbon flow from plants to soil and contributing to improvements in the global soil carbon pool (Hawkins *et al.*, 2023; Wang *et al.*, 2023; Kakouridis *et al.*, 2024).

As of April 2024, 355 fungal species have been classified into 5 classes and 44 genera, which are listed as AM fungi (http://www.amf-phylogeny.com/index.html) belonging to *Glomeromycota*. These species’ functions and life histories differ according to the strain (Chagnon *et al.*, 2013), and several culture collections of AM fungi have been established worldwide (Berruti *et al.*, 2014). In Japan, the National Agricultural Research Organization (NARO) Genebank project (https://www.gene.affrc.go.jp/index_en.php) maintains pure cultures of several AM fungal strains and distributes them for research and educational purposes upon request.

Prior to the submission of a newly isolated fungal strain to the NARO Genebank, it needs to be identified. Although rRNA gene sequences are commonly used for the molecular identification of organisms, unique difficulties are associated with AM fungi due to the high number of polymorphisms in their rRNA sequences (Maeda *et al.*, 2018) and their multinuclear nature (Trouvelot *et al.*, 1999; Denison and Kiers, 2011). One widely accepted method for the molecular classification of AM fungi is sequencing the so-called Krüger’s fragment (Krüger *et al.*, 2009; Krüger *et al.*, 2012), which includes partial SSU, ITS, and partial LSU regions. This fragment is approximately 1.5‍ ‍kb in length and cannot typically be sequenced in a single run using Sanger sequencing. Multiple overlapping sequences need to be obtained and concatenated to reconstruct the entire region. Furthermore, cloning is essential because PCR products from a single isolate of AM fungi often contain a mixture of sequence variants, making direct sequencing unreliable, unlike in many other fungal species. However, there are no established guidelines on how many sequences need to be analyzed in order to achieve reliable identification at the strain level. Growing consensus is that rRNA gene sequences alone are not sufficient to identify AM fungi at the species level because variations in the region derived from a single spore may exceed the threshold of 3% typically used for species differentiation, and also some rRNA gene loci may cluster across species boundaries in phylogenetic anal­yses (Oliveira *et al.*, 2024; Corradi *et al.*, 2025).

I previously reported a simple identification method of AM fungal cultures using PCR-denaturing gradient gel electrophoresis (DGGE) (Ohtomo *et al.*, 2019). Our findings showed that different isolates of *Gigaspora margarita* exhibited distinct DGGE banding patterns, whereas multiple spores from the same isolate yielded identical patterns. These results suggest the potential of rRNA polymorphisms as a useful marker for strain-level identification in some AM fungal species.

Recent advances in high-throughput sequencing technologies, such as Illumina MiSeq, have enabled detailed comparisons of microbial communities based on operational taxonomic units (OTUs) and, more recently, amplicon sequence variants (ASVs) (Van Geel *et al.*, 2014; Nilsson *et al.*, 2019). Therefore, each AM fungal isolate may be characterized by its unique composition of ASVs reflecting intra-isolate polymorphisms in rRNA genes, which will enable both species- and strain-level identification. Unlike conventional approaches that consider rRNA gene heterogeneity as an obstacle, it was utilized herein as a source of taxonomic information. To the best of our knowledge, this is the first study to apply intra-isolate rRNA polymorphism patterns to AM fungal strain identification. Illumina MiSeq sequencing was used to examine the ASV profiles of cultured AM fungal isolates and evaluate their utility for taxonomic identification.

## Materials and Methods

### Preparation of DNA templates from AM fungal spores

The spores of AM fungal isolates were obtained from the NARO Genebank, Japan (https://www.gene.affrc.go.jp/about_en.php). Thirty-three isolates consisting of 9 different genera used in this study are shown in Table 1. Spores were isolated from cultures, which were proliferated in 2022 using Bahia grass and white clover as hosts, by wet sieving and sucrose gradient centrifugation. Kaneka Easy DNA Extraction kit ver. 2 (Kaneka, Tokyo, Japan) was employed for DNA extraction from isolated spores. In brief, spores were crushed in a BioMasher II disposable homogenizer tube (Nippi, Tokyo, Japan) containing 30‍ ‍μL Solution A, and 25‍ ‍μL of the mixture was transferred to a 200-μL PCR tube. The tubes were heated at 98°C in the thermal cycler for 8‍ ‍min, and 3.5‍ ‍μL Solution B was added with vortexing after cooling down. The extract was centrifuged at 18,000×*g* for 3‍ ‍min, and the supernatant was recovered and used as a PCR template.

### Sequencing

The partial sequences of the rRNA region were amplified using 5 different primer sets listed in Table S1 and then subjected to Illumina MiSeq sequencing (Illumina). Due to the limited amount of the DNA template, *Rhizophagus intraradices* Habte (MAFF520059) was analyzed with three primer sets, AMV4.5NF/AMVR, FLd3-1/FLR2, and ITS1-F_KYO1/ITS2_KYO2. KOD-One PCR Master Mix (TOYOBO) was used for amplification. After purification with 0.8 vol of AMPure XP beads (Beckman Coulter), amplicons were indexed with Nextera XT Index Kit v2 (Illumina) and sequenced using the MiSeq platform with the MiSeq Reagent Kit v3 (600-cycle) (Illumina) at the Institute of Crop Science, NARO. The paired-end reads obtained were processed using the QIIME2 pipeline (version 2020.2; Bolyen *et al.*, 2019), including the merging of forward and reverse reads, filtering to remove low-quality sequences with DADA2, and taxonomic classification. In the classification of ASVs, classifiers pre-trained with the SILVA database (release 138.1; Quast *et al.*, 2013) for the AMV4.5NF/AMVR and 1422F/1642R amplicons and the UNITE database (QIIME release ver. 9; Abarenkov *et al.*, 2023) for ITS1-F_KYO1/ITS2_KYO2, gITS7/ITS4 and FLd3-1/FLR2 amplicons were used.

### Bioinformatics

Before proceeding, minor ASVs with read counts accounting for less than 1% of all reads in each sample were removed. The R package “phyloseq” (McMurdie and Holmes, 2013) was used to remove non-AM sequences and for rarefaction and weighted UniFrac (wUF) distance calculations, while the package “cluster” was used for a cluster anal­ysis with the Unweighted Pair Group Method with Arithmetic Mean (UPGMA). In the rarefaction step, samples with less than 3,000 reads remaining after the removal of non-AM sequences were excluded.

Phylogenetic trees of the selected ASVs and reference sequences were constructed using MEGA11 (Stecher *et al.*, 2020; Tamura *et al.*, 2021). Reference sequences were selected from the classifier database used in the QIIME2 classification above based on clear species-level assignments. Sequences were aligned using the ClustalW algorithm, and phylogenetic trees were generated using the maximum-likelihood method and Tamura-Nei model (Tamura and Nei, 1993).

### Data availability

MiSeq data reported in this study, except for those of *Ambispora leptoticha* KM-2 and *Archaeospora trappei* SA1-1, have been deposited in the DNA Data Bank of Japan (DDBJ). These two strains were excluded from the registration because it was not possible to provide source strain information due to a discrepancy between the previous taxonomic classification and current identification based on MiSeq sequencing. Furthermore, spore morphology suggested that the strains may not have been properly maintained during subculturing, and also that other AM fungi may have proliferated instead. The corresponding BioSample accession numbers for each fungal strain–primer set combination are provided in Table S2.

## Results

### MiSeq sequencing

Although the amplification efficiency and required PCR cycles varied among samples, all spore DNA samples showed effective amplification with the five primer sets used in this study. Read numbers of more than 12,000 per sample were obtained, and more than 90% of input reads remained after DADA2 quality filtering for all samples (data not shown).

### Taxonomic classification of ASVs

Table 1 summarizes the ASV profile of each strain, as elucidated using the five PCR primer sets, and the corresponding ASV classifications. With a few exceptions, most strains exhibited ASVs consistent with the taxonomic identification of the strain. For example, ASVs detected from three *Acaulospora* isolates were all, except one, identified as *Acaulospora* sequences, although the number of ASVs detected in each isolate varied from 1 to 10 depending on the strain and primer set used. Strain SA1-1 previously identified as *Ar. trappei* contained ASVs classified as *Paraglomus* and was subsequently re-identified as *Paraglomus* based on spore morphological characteristics (data not shown). Among the strains tested, those belonging to *Gi. margarita* were highly polymorphic, showing more than 10 ASVs when amplified with AMV4.5NF/AMVR, ITS1-F_KYO1/ITS2_KYO2, and FLd3-1/FLR2. Among the five primer sets tested, the region amplified with 1422F/1642R exhibited the lowest level of polymorphism in most strains, except *Gi. margarita*.

In most of the isolates, ASVs classified as non-AM accounted for less than 1% of the total reads. However, for gITS7/ITS4 amplicons of *Cetraspora pellucida* AM-3, *Ce. pellucida* SZ-3, and *Dentiscutata cerradensis* TK-1, non-AM ASVs accounted for more than 70% of all reads. Therefore, these samples were excluded from subsequent anal­yses.

After rarefying to an even reading depth, rarefaction curves plateaued at a relatively low read number of a few hundred reads (Fig. S1), suggesting that the sequencing depth obtained was sufficient to capture the actual ASV diversity in each isolate.

### Clustering of AM fungal isolates based on ASV composition

**Fig. 1.**
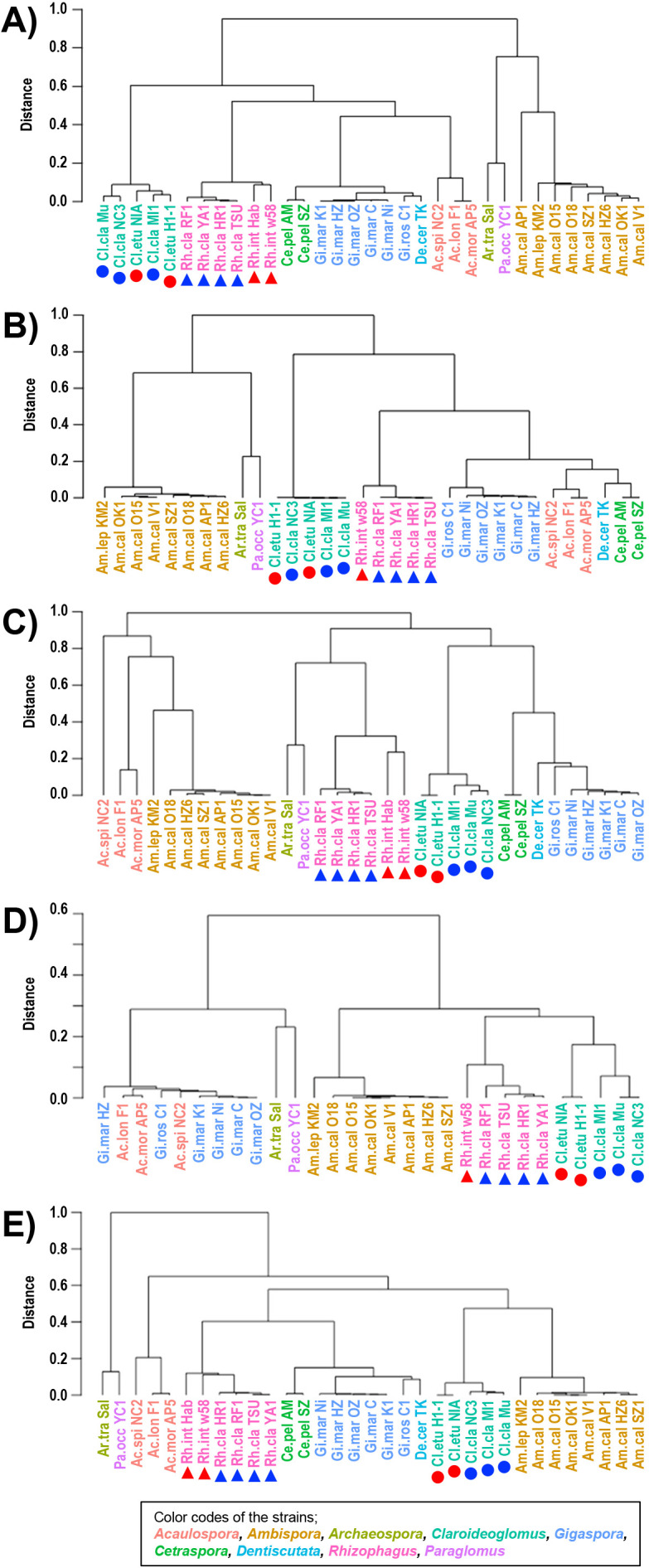
Construction of dendrograms of AM fungal strains based on weighted UniFrac distances. The primer sets used to obtain ASVs were as follows: AMV4.5NF/AMVR (a), 1422F/1642R (b), ITS1-F_KYO1/ITS2_KYO2 (c), gITS7/ITS4 (d), and FLd3-1/FLR2 (e). Strain abbreviations are shown in Table 1. Color coding of strain names represents genus-level grouping. Species of *Rhizophagus* and *Claroideoglomus* were marked as follows: *Rh. clarus* and *Rh. intraradices*, by blue and red triangles, and *Cl. claroideum* and *Cl. etunicatum* by blue and red circles, respectively. Abbreviations and color codes for each strain were used in subsequent figures.

Fig. 1 shows the results of a cluster anal­ysis based on wUF distances among strains analyzed using the same primer sets. As indicated by the strain color codes, strains belonging to the same genus generally clustered together, suggesting that the method successfully distinguished among different species irrespective of the primer sets used. One exception was that *Acaulospora* and *Gigaspora* strains clustered together when amplified with gITS7/ITS4 (Fig. 1D). At the species level, two *Rhizophagus* strains, *Rh. clarus* and *Rh. intraradices* were separated into different sub-clusters regardless of the primer sets used. In contrast, *Claroideoglomus claroideum* and *Cl. etunicatum* were not successfully separated when the primer sets AMV4.5NF/AMVR and 1422F/1642R were used.

### Phylogenetic anal­ysis of ASVs

**Fig. 2.**
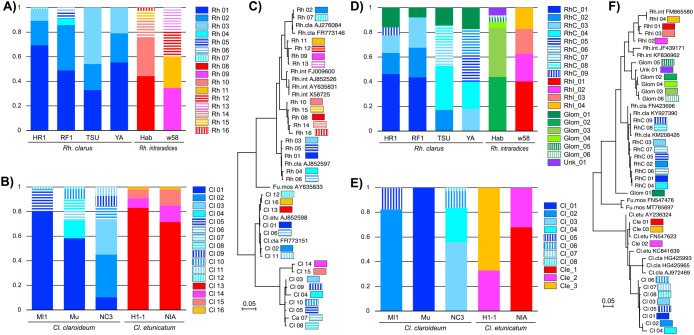
ASV compositions and phylogenetic relationships of six *Rhizophagus* strains (four *Rh. clarus* and two *Rh. intraradices*) and five *Claroideoglomus* strains (three *Cl. claroideum* and two *Cl. etunicatum*). Panels a and d correspond to *Rhizophagus*, b and e to *Claroideoglomus*, and c and f show the phylogenetic trees of ASVs and sequences of some reference strains. Panels a, b, and c indicate the results obtained using the AMV4.5NF/AMVR primer set, while panels d, e, and f indicate those obtained using ITS1-F_KYO1/ITS2_KYO2. Abbreviations for ASVs are as follows: Rh, *Rhizophagus*; Cl, *Claroideoglomus*; RhC, *Rh. clarus*; RhI, *Rh. intraradices*; Glom, *Glomerales*; Cle, *Cl. etunicatum*; Unk, unknown AM fungi.

The phylogenetic relationships of ASVs were investigated to elucidate the cause of the different taxonomic resolution observed among the primer sets. The ASV composition of six *Rhizophagus* strains and five *Claroideoglomus* strains, obtained using the AMV4.5NF/AMVR and ITS1-F_KYO1/ITS2_KYO2 primer sets, were compared in Fig. 2A and B and in Fig. 2D and E, respectively. The phylogenetic trees of the ASVs, together with some reference sequences, are shown in Fig. 2C and F. Bar charts showing the ASV composition indicate that different species did not share any ASVs when using either primer set. In the case of *Rhizophagus*, ASVs derived from different species were found to be phylogenetically distinguished from each other with both primer sets (Fig. 2C and F). In contrast, some of the ASVs derived from different *Claroideoglomus* species obtained with the AMV4.5NF/AMVR primer set were grouped into the same clades (Fig. 2C), whereas those amplified with the ITS1-F_KYO1/ITS2_KYO2 primer set were separated into distinct clades (Fig. 2F). This limited phylogenetic separation likely contributed to the reduced resolution of the clustering anal­ysis for this genus when using AMV4.5NF/AMVR primer sets.

## Discussion

The strain-level identification of AM fungi remains challenging due to their multinucleate nature and the high number of polymorphisms in rRNA regions within a single genome. Therefore, single rRNA reads from an isolate are often insufficient to accurately distinguish between strains. However, there is still no clear consensus on how many rRNA sequences from a single isolate are necessary for reliable identification. There is a growing consensus that the taxonomic classification of AM fungi ideally requires comprehensive genomic information, rather than relying solely on rRNA gene sequences because intra-isolate variations in rRNA genes may exceed the 3% divergence threshold commonly used for species delimitation (Oliveira *et al.*, 2024; Corradi *et al.*, 2025).

Recent improvements in high-throughput sequencing technologies have enabled us to perform comparative anal­yses of gene assemblies. The present study aimed to apply these advances in molecular ecology to characterize individual AM fungal isolates in terms of their population-level genetic composition. This approach enabled not only species-level identification, but in some cases strain-level identification, thereby extending the utility of rRNA gene data beyond traditional taxonomic resolution.

### MiSeq sequencing and taxonomic classification of ASVs

The number and composition of ASVs detected from the same AM fungal isolate varied depending on the primer pair used for amplification. Among the five primer sets tested, 1422F/1642R showed the least variability, reflecting the 18S rRNA region it targets being more conserved than the ITSs or 28S regions (*e.g.*, Maeda *et al.*, 2018). The extent of ASV diversity also varied among different AM fungal isolates. This variation likely reflects biological differences, such as the number of rRNA gene copies and the degree of intra-genomic heterogeneity among strains (Oliveira *et al.*, 2024). Among the strains tested, *Gi. margarita* exhibited the highest viability with more than 10 ASVs when analyzed with‍ ‍AMV4.5NF/AMVR, ITS1-F_KYO1/ITS2_KYO2, and FLd3/FLR2 (Table 1). These results suggest that the primer choice and fungal strain both significantly affect ASV profiles.

Rather than treating these differences as technical artifacts, the ASV composition (resulting from both primer-dependent detection and strain-specific polymorphisms) may serve as a unique “fingerprint” for each AM fungal isolate. This approach provides a practical framework for identifying and comparing AM fungal strains, particularly in the context of culture collections.

However, when considering the multinucleate nature of AM fungi with single spores containing thousands of nuclei as well as high polymorphisms in their rRNA regions, the overall number of ASVs detected from AM fungal isolates was low, with at most 20 variants per strain (Table 1). The rarefaction anal­ysis showed that the number of ASVs reached a plateau with a relatively small reading depth of a few hundred reads (Fig. S1), which also indicated fewer intra-isolate variations in rRNA regions than expected. This result is consistent with the findings reported by Lin *et al.* (2014) showing that the genome sequence of several nuclei isolated from a single strain of the AM fungus *Rh. irregularis* was highly similar. Their *de novo* genome assemblies revealed minimum polymorphisms among the nuclei, although the 45S rRNA gene sequence within a single genome showed pronounced divergence, supporting the homokaryotic nature of this species.

In the present study, a stringent cut-off value of 1% was applied to remove rare reads. This approach was selected because when using a 0.1% cut-off, a significant increase in non-AM fungal reads was observed. This may be related to the use of soil-derived spores without surface sterilization for DNA extraction, and suggests that improved DNA template preparation and the exclusion of non-AM sequences may be important for enhancing the accuracy of subsequent taxonomic anal­yses.

### Characterization of AM fungal isolates based on the ASV composition

The cluster anal­ysis indicated that the ASV composition was effective for the identification of AM fungi, at least at the genus level. Strains belonging to the same genus generally clustered together irrespective of the primer sets used (Fig. 1). However, some exceptions were observed. For example, *Acaulospora* strains were not clearly separated from *Ambispora* strains when amplified with ITS1-F_KYO1/ITS2_KYO2 (Fig. 1C), and they clustered together with *Gigaspora* when the gITS7/ITS4 primer set was used (Fig. 1D). The reasons for these inconsistencies remain unclear. Notably, no common ASVs were shared among *Acaulospora*, *Ambispora*, and *Gigaspora* (Table 1), suggesting that the clustering patterns observed were not due to shared sequence variants. In the preliminary anal­ysis that included ASVs with an unknown taxonomic classification (such as those labeled “Fungi_phy_Incertae_sedis” or unassigned at the phylum level, which were detected in isolates of *Cetraspora* or *Dentiscutata*), these genera were more clearly separated into distinct clusters. Although unassigned ASVs were not detected in *Acaulospora*, *Ambispora*, or *Gigaspora* isolates, their inclusion appeared to affect the overall cluster structure, possibly through indirect effects on distance metrics. Further data accumulation from additional isolates is expected to improve the resolution and reliability of the taxonomic assignments based on ASV profiles.

Although three *Cl. claroideum* and two *Cl. etunicatum* strains were not separated in the cluster anal­ysis using AMV4.5NF/AMVR (Fig. 1A), the ASV composition revealed that no ASV was shared between these two species. The ASV commonly found in the two *Cl. etunicatum* (Cl 13 in Fig. 2B) was phylogenetically close (Fig. 2C) to the main ASVs of *Cl. claroideum* (Cl 01 and 02 in Fig. 2B), which may explain why the two species were not separated in the cluster anal­ysis based on wUF distances.

## Conclusion and future prospects

The present results indicate that the species-level identification of AM fungi is possible using MiSeq. This approach also appears to be effective for the strain-level differentiation of AM fungal isolates because an ASV composition anal­ysis revealed some strain-specific ASVs. The accumulation of MiSeq data on AM fungal isolates will be valuable from the viewpoint of enhancing public databases and improving taxonomic resolution in future studies; for example, ASVs classified as *Glomeraceae* and unidentified *Glomeromycota* were confirmed to be derived from *Rh. intraradices* in the present study (Fig. 2D and F).

Although AM fungi have long been considered asexual and proliferate clonally, previous studies suggested that they exchange genetic material via hyphal fusion (Croll *et al.*, 2009). AM fungal strains of the same species that do not share any common ASVs, such as the two *Rh. intraradices* and three *Cl. claroideum* strains, which exhibited no shared ASVs when amplified with ITS1-F_KYO1/ITS2_KYO2 (Fig. 2D and E), may serve as valuable models for investigating this phenomenon. Co-culturing these strains and observing the emergence of isolates harboring ASVs from both parental lines may provide direct evidence for genetic interactions among isolates.

Therefore, clarifying the ASV composition of each strain is not only valuable for accurate taxonomic identification, but also offers a promising tool to examine the mechanisms underlying genetic interactions in these fungi. To establish MiSeq-based ASV profiles as reliable taxonomic indicators for AM fungi, it will be essential to accumulate corres­ponding data from a wider range of isolates. Additionally, verifying the stability of the ASV composition across fungal generations will be important for ensuring the robustness of this approach.

## Citation

Ohtomo, R. (2025) Identification of Arbuscular Mycorrhizal Fungal Isolates using MiSeq Sequencing. *Microbes Environ ***40**: ME25040.

https://doi.org/10.1264/jsme2.ME25040

## Supplementary Material

Supplementary Material

## Figures and Tables

**Table 1.** Strains used in this study and their ASV profiles

Strain Used				Detected ASVs^2)3)4)^				
Identification	Label	MAFF No.^1)^		AMV4.5NF	1422F	ITS1-F_KYO1	gITS7	FLd3
***Acaulospora***								
*Ac. longula* F-1	Ac.lon F1	520060		Ac (2)	Ac (1)	Ac (6)	Ac (7)	Ac (6)
*Ac. morrowiae* AP-5	Ac.mor AP5	520081		Ac (3)	Ac (1)	Ac (6)	Ac (7)	Ac (6)
*Ac. spinosa* NC-2	Ac.spi NC2	520094		Ac (6)	Ac (2)	Ac (4)	Ac (8) **No (1)**	Ac (10)
***Ambispora***								
*Am. callosa* AP-1	Am.cal AP1	520080		Am (4) **Gi (7)**	Am (2)	Am (7)	Am (7)	Am (6)
*Am. callosa* HZ-6k	Am.cal HZ6	520073		Am (6) **Ac (1)**	Am (2)	Am (7)	Am (8)	Am (7)
*Am. callosa* OK-1	Am.cal OK1	520057		Am (6) **Rh (1)**	Am (3)	Am (7) **No (1)**	Am (7)	Am (8)
*Am. callosa* OK-15	Am.cal O15	520072		Am (6) **Ac (1) Gi (2)**	Am (3)	Am (7)	Am (7)	Am (7)
*Am. callosa* OK-m18	Am.cal O18	520077		Am (7) **Rh (2)**	Am (3)	Am (6) **No (1)**	Am (6) **No (1)**	Am (7)
*Am. callosa* SZ-1	Am.cal SZ1	520084		Am (5)	Am (4)	Am (5) **No (1)**	Am (8)	Am (7)
*Am. callosa* V-1	Am.cal V1	520058		Am (6) **Gi (2)**	Am (3)	Am (7)	Am (7)	Am (8)
*Am. leptoticha* KM-2	Am.lep KM2	520090		Am (7) **Gi (1) Pa (1)**	Am (4) **Pa (1)**	Am (7) **Pa (1)** Un (1)	Am (8) AMF (1)	Am (11) **Pa (1)**
***Archaeospora***								
*Ar. trappei* SA1-1	Ar.tra Sal	520075		**Ra (1) Pa (1)**	Pa (1) **No (2)**	**Pa (1)**	AMF (2)	**Pa (2)**
***Claroideoglomus***								
*Cl. claroideum* MI-1	Cl.cla MI1	520092		Cl (3)	Cl (1)	Cl (2)	Cl (5)	Cl (4) Un (2)
*Cl. claroideum* Mu-243	Cl.cla Mu	520061		Cl (6)	Cl (1) **No (1)**	Cl (1)	Cl (4)	Cl (5) Un (3)
*Cl. claroideum* NC3	Cl.cla NC3	520097		Cl (6)	Cl (1) AMF (1)	Cl (5)	Cl (3)	Cl (2) Un (2)
*Cl. etunicatum* H1-1	Cl.etu H1-1	520053		Cl (4)	Cl (1)	Cl (2)	Cl (2)	Cl (1) Un (2)
*Cl. etunicatum* NIAES	Cl.etu NIA	520082		Cl (4)	Cl (1)	Cl (2)	Cl (2)	Cl (1) Un (2)
***Gigaspora***								
*Gi. margarita* C	Gi.mar C	520054		Gi (12)	Gi (7) Div (2)	Gi (13)	Gi (5)	Gi (17)
*Gi. margarita* HZ-4e	Gi.mar HZ	520074		Gi (11)	Gi (6) Div (2)	Gi (14)	Gi (5)	Gi (16)
*Gi. margarita* K-1	Gi.mar K1	520052		Gi (11)	Gi (8) Div (1)	Gi (13)	Gi (7)	Gi (16)
*Gi. margarita* Ni-A	Gi.mar Ni	140115		Gi (12)	Gi (7) Div (1)	Gi (13)	Gi (6)	Gi (18)
*Gi. margarita* OZ-2	Gi.mar OZ	520085		Gi (11) Div (1)	Gi (6) Div (2)	Gi (14)	Gi (4)	Gi (16)
*Gi. rosea* C1	Gi.ros C1	520062		Gi (7)	Gi (2) Div (2)	Gi (4) Div (1)	Gi (6) **De (1)** Un (1) **No (1)**	Gi (8)
***Cetraspora***								
*Ce. pellucida* AM-3	Ce.pel AM	520079		**Ra (4)** Div (2)	Ce (3)	Ce (6)	Ce (1) **No (2)**	Ce (11) Div (4)
*Ce. pellucida* SZ-3	Ce.pel SZ	520083		**Gi (2) Ra (1)** Div (1)	Ce (1) **No (2)**	Ce (3) Fu (2)	Ce (4) Un (1) **No (5)**	Ce (4) Div (3)
***Dentiscutata***								
*De. cerradensis* TK-1	De.cer TK	520056		De (3) **Gi (5)**	De (2) **Gi (1)** Div (2) **No (1)**	De (2) **Gi (3)**	Sc (2) Div (1) **No (1)**	De (3) Div (1) **Gi (8) No (1)**
***Rhizophagus***								
*Rh. clarus* HR1	Rh.cla HR1	520076		Rh (4)	Rh (2)	Rh (4) Gl (1)	Gl (4) AMF (1)	Rh (2) AMF (5)
*Rh. clarus* RF1	Rh.cla RF1	520086		Rh (5)	Rh (2) **No (2)**	Rh (3) Gl (1)	Gl (1) **No (2)**	Rh (3) Gl (1) AMF (4) **No (1)**
*Rh. clarus* TSU-2	Rh.cla TSU	520089		Rh (3)	Rh (2)	Rh (4) Gl (1)	Gl (3) AMF (1)	Rh (1) Gl (1) AMF (4)
*Rh. clarus* YA-1	Rh.cla YA1	520096		Rh (3)	Rh (2)	Rh (4) Gl (1) **No (1)**	Gl (4) AMF (1)	Rh (1) Gl (2) AMF (2)
*Rh. intraradices* Habte	Rh.int Hab	520059		Rh (5)	—	Gl (5) AMF (1)	—	Gl (5) AMF (1)
*Rh. intraradices* w5845	Rh.int w58	520088		Rh (4)	Rh (1) Gl (1) **No (2)**	Rh (4)	Rh (5)	Rh (4) Gl (1)
***Paraglomus***								
*Pa. occultum* YC-1	Pa.occ YC1	520091		Pa (1)	AMF (1) **No (1)**	Pa (2)	Pa (3)	Pa (2)

1) Accession numbers of strains in the NARO Genebank. 2) List of ASVs and their classification obtained with each primer set (only the forward primer is shown). 3) Abbreviations used in the ASV classification: Ac, Acaulospora; Am, Ambispora; Cl, Claroideoglomus; Ce, Cetraspora; De, Dentiscutata; Gi, Gigaspora; Pa, Paraglomus; Ra, Racocetra; Rh, Rhizophagus; Sc, Scutellospora; Gl, Glomerales; Div, Diversisporaceae; AMF, unknown Glomeromycota; Fu, unknown Fungi; No, non-Glomeromycota; Un, unassigned; —, not analyzed. 4) Notation: Underlining indicates the classification above the genus level. Numbers in parentheses represent the number of distinct ASVs detected. Bold text indicates an inconsistency between the ASV classification and strain identification.
