# Supplementary material for: Identification of Arbuscular Mycorrhizal Fungal Isolates using MiSeq Sequencing
Source: Microbes Environ. 2025 Nov 14;40(4):ME25040. doi: 10.1264/jsme2.ME25040 (PMC12727196; doi:10.1264/jsme2.ME25040)
Supplement: Supplementary file 1 — Supplementary Material [file 40_25040_s1.pdf]

Table S1, Primer sets used in this study

Forward primer	Reverse primer	Target	References
AMV4.5NF	AMVR	18S V4-V5	Morimoto et al., 2018; Sato et al., 2005
FLd3-1	FLR2	28S D2	Niwa et al., 2018
1422F	1642R	18S V9	Wang et al., 2014
ITS1-F_KYO1	ITS2_KYO2	ITS1	Toju et al., 2012
gITS7	ITS4	ITS2	Ihrmark et al., 2012

(References)

1. Ihrmark, K., I.T.M. Bdeker, K. Cruz-Martinez, H. Friberg, A. Kubartova, J. Schenck, Y. Strid, J. Stenlid, M. Brandström-Durling, K.E. Clemmensen, and B.D. Lindahl (2012) New primers to amplify the fungal ITS2 region - evaluation by 454-sequencing of artificial and natural communities. FEMS Microbiol Ecology. 82:666-677.
2. Morimoto, S., T. Uchida, H. Matsunami, and H. Kobayashi (2018) Effect of winter wheat cover cropping with no-till cultivation on the community structure of arbuscular mycorrhizal fungi colonizing the subsequent soybean. Soil Sci Plant Nutr. 64:545-553.
3. Niwa, R., T. Koyama, T. Sato, K. Adachi, K. Tawaraya, S. Sato, H. Hirakawa, S. Yoshida, and T. Ezawa (2018) Dissection of niche competition between introduced and indigenous arbuscular mycorrhizal fungi with respect to soybean yield responses. Scientific Reports. 8:7419.
4. Sato, K., Y. Suyama, M. Saito, and K. Sugawara (2005) A new primer for discrimination of arbuscular mycorrhizal fungi with polymerase chain reaction-denature gradient gel electrophoresis. Grassl Sci. 51:179-181.
5. Toju, H., A.S. Tanabe, S. Yamamoto, and H. Sato (2012) High-coverage ITS primers for the DNA-based identification of ascomycetes and basidiomycetes in environmental samples. PLoS One. 7:e40863.
6. Wang, Y., R.M. Tian, Z.M. Gao, S. Bougouffa, and P.Y. Qian (2014) Optimal eukaryotic 18S and universal 16S/18S ribosomal RNA primers and their application in a study of symbiosis. PLoS One. 9:e90053.

Table S2. DDBJ BioSample accession numbers obtained in this study

Strain	BioSample Accession Number				
	AMV4.5NF	1422F	ITS1-F_KYO1	gITS7	FLd3
Acaulospora					
Ac. longula F-1	SAMD00827510	SAMD00827510	SAMD00827510	SAMD00827510	SAMD00827510
Ac. morrowiae AP-5	SAMD00827511	SAMD00827511	SAMD00827511	SAMD00827511	SAMD00827511
Ac. spinosa NC-2	SAMD00827512	SAMD00827512	SAMD00827512	SAMD00827512	SAMD00827512
Ambispora					
Am. callosa AP-1	SAMD00827513	SAMD00827513	SAMD00827513	SAMD00827513	SAMD00827513
Am. callosa HZ-6k	SAMD00827514	SAMD00827514	SAMD00827514	SAMD00827514	SAMD00827514
Am. callosa OK-1	SAMD00827515	SAMD00827515	SAMD00827515	SAMD00827515	SAMD00827515
Am. callosa OK-15	SAMD00827516	SAMD00827516	SAMD00827516	SAMD00827516	SAMD00827516
Am. callosa OK-m18	SAMD00827517	SAMD00827517	SAMD00827517	SAMD00827517	SAMD00827517
Am. callosa SZ-1	SAMD00827518	SAMD00827518	SAMD00827518	SAMD00827518	SAMD00827518
Am. callosa V-1	SAMD00827519	SAMD00827519	SAMD00827519	SAMD00827519	SAMD00827519
Claroideoglomus					
Cl. claroideum MI-1	SAMD00827520	SAMD00827520	SAMD00827520	SAMD00827520	SAMD00827520
Cl. claroideum Mu-243	SAMD00827521	SAMD00827521	SAMD00827521	SAMD00827521	SAMD00827521
Cl. claroideum NC3	SAMD00827522	SAMD00827522	SAMD00827522	SAMD00827522	SAMD00827522
Cl. etunicatum H1-1	SAMD00827523	SAMD00827523	SAMD00827523	SAMD00827523	SAMD00827523
Cl. etunicatum NIAES	SAMD00827524	SAMD00827524	SAMD00827524	SAMD00827524	SAMD00827524
Gigaspora					
Gi. margarita C	SAMD00827525	SAMD00827525	SAMD00827525	SAMD00827525	SAMD00827525
Gi. margarita HZ-4e	SAMD00827526	SAMD00827526	SAMD00827526	SAMD00827526	SAMD00827526
Gi. margarita K-1	SAMD00827527	SAMD00827527	SAMD00827527	SAMD00827527	SAMD00827527
Gi. margarita Ni-A	SAMD00827528	SAMD00827528	SAMD00827528	SAMD00827528	SAMD00827528
Gi. margarita OZ-2	SAMD00827529	SAMD00827529	SAMD00827529	SAMD00827529	SAMD00827529
Gi. rosea C1	SAMD00827530	SAMD00827530	SAMD00827530	SAMD00827530	SAMD00827530
Cetraspora					
Ce. pellucida AM-3	SAMD00827540	SAMD00827540	SAMD00827540	SAMD00827540	SAMD00827540
Ce. pellucida SZ-3	SAMD00827541	SAMD00827541	SAMD00827541	SAMD00827541	SAMD00827541
Dentiscutata					
De. cerradensis TK-1	SAMD00827539	SAMD00827539	SAMD00827539	SAMD00827539	SAMD00827539
Rhizophagus					
Rh. clarus HR1	SAMD00827532	SAMD00827532	SAMD00827532	SAMD00827532	SAMD00827532
Rh. clarus RF1	SAMD00827533	SAMD00827533	SAMD00827533	SAMD00827533	SAMD00827533
Rh. clarus TSU-2	SAMD00827534	SAMD00827534	SAMD00827534	SAMD00827534	SAMD00827534
Rh. clarus YA-1	SAMD00827535	SAMD00827535	SAMD00827535	SAMD00827535	SAMD00827535
Rh. interaradices Habte	SAMD00827536		SAMD00827536		SAMD00827536
Rh. interaradices w5845	SAMD00827537	SAMD00827537	SAMD00827537	SAMD00827537	SAMD00827537
Paraglomus					
Pa. occultum YC-1	SAMD00827531	SAMD00827531	SAMD00827531	SAMD00827531	SAMD00827531

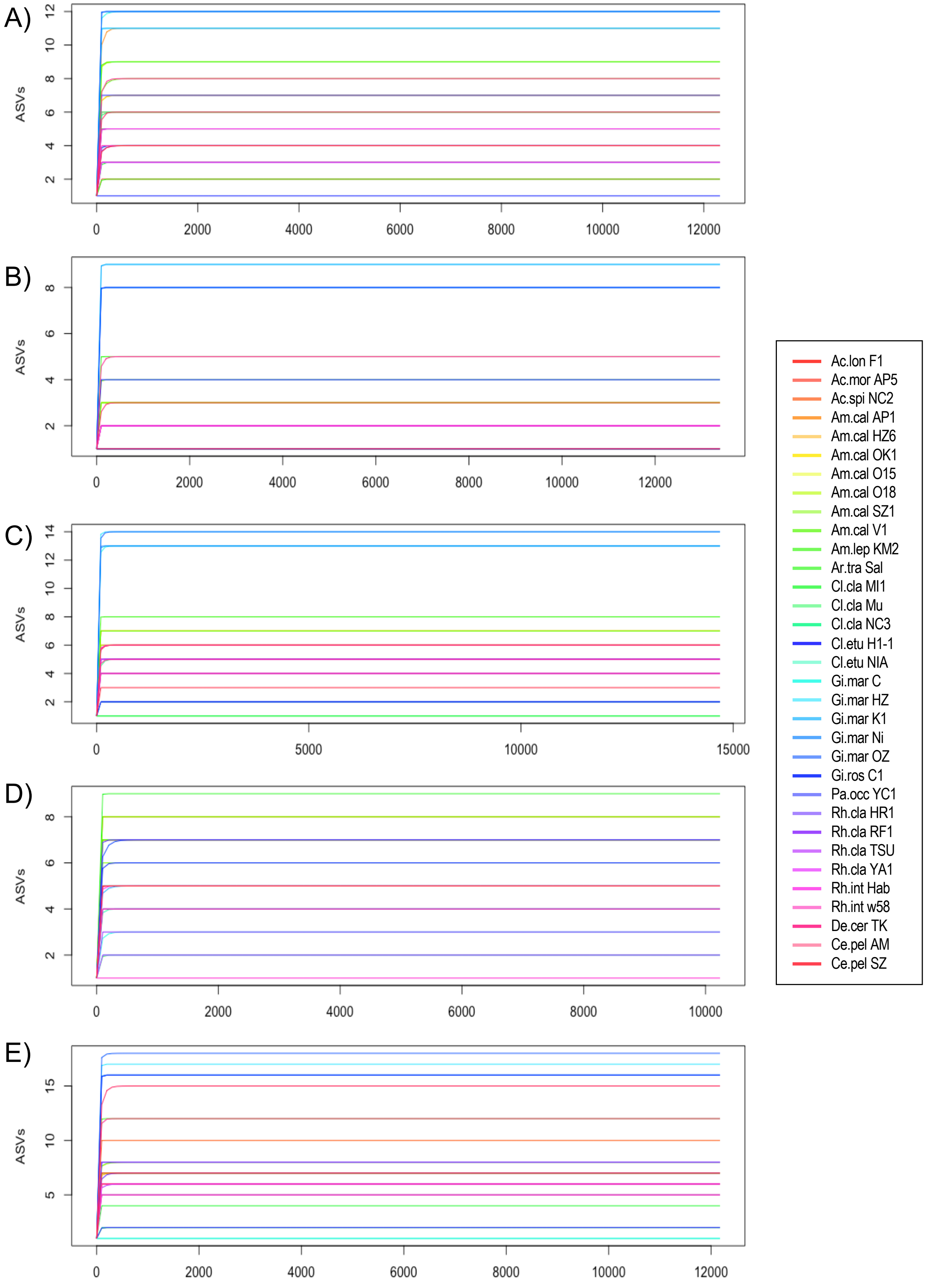

Figure S1

Rarefaction curves of MiSeq data in this study. The primer set used to obtain ASVs was as follows: AMV4.5NF/AMVR (a), 1422F/1642R (b), ITS1-F_KYO1/ITS2_KYO2 (c), gITS7/ITS4 (d), and FLd3-1/FLR2 (e).
